# Cryptic Transcription and Early Termination in the Control of Gene Expression

**DOI:** 10.4061/2011/653494

**Published:** 2011-11-24

**Authors:** Jessie Colin, Domenico Libri, Odil Porrua

**Affiliations:** ^1^LEA Laboratory of Nuclear RNA Metabolism, Centre de Génétique Moléculaire (CNRS), UPR3404, 1 Avenue de la Terrasse, 91190 Gif sur Yvette, France; ^2^Centre for mRNP Biogenesis and Metabolism, Aarhus University, C.F. Møllers Alle, Building 130, 8000 Aarhus C, Denmark

## Abstract

Recent studies on yeast transcriptome have revealed the presence of a large set of RNA polymerase II transcripts mapping to intergenic and antisense regions or overlapping canonical genes. Most of these ncRNAs (ncRNAs) are subject to termination by the Nrd1-dependent pathway and rapid degradation by the nuclear exosome and have been dubbed cryptic unstable transcripts (CUTs). CUTs are often considered as by-products of transcriptional noise, but in an increasing number of cases they play a central role in the control of gene expression. Regulatory mechanisms involving expression of a CUT are diverse and include attenuation, transcriptional interference, and alternative transcription start site choice. This review focuses on the impact of cryptic transcription on gene expression, describes the role of the Nrd1-complex as the main actor in preventing nonfunctional and potentially harmful transcription, and details a few systems where expression of a CUT has an essential regulatory function. We also summarize the most recent studies concerning other types of ncRNAs and their possible role in regulation.

## 1. Introduction

The development of new technologies in the field of transcriptome analysis has revealed an unexpected level of complexity in the eukaryotic transcription landscape. High-resolution techniques as tiling arrays and, more recently, RNA deep-sequencing have shown that a large proportion of transcripts are not associated to well-defined functional units as genes, rRNA, tRNA, and so forth, giving rise to the concepts of “pervasive” and “hidden” transcription [1, 2]. Those transcripts are often rapidly degraded, so that they remain invisible unless RNA degradation is prevented, for example, by inactivation of the degradation machinery. 

Recent deep sequencing of nascent transcripts [3] has allowed a more direct analysis of RNA polymerase distribution in wild-type yeast cells obviating the need for working in mutants of the degradation pathway. These experiments have nicely confirmed the existence of hidden transcription.

In the yeast *Saccharomyces cerevisiae*, the main class of non-coding unstable RNAs transcribed by the RNA polymerase II is constituted by the Cryptic Unstable Transcripts (CUTs). CUTs are capped, relatively small, with an average length of 200 to 500 bp and with heterogeneous 3′ ends [4]. Their transcription is terminated by a pathway dependent upon the Nrd1 complex (see below), which targets them for polyadenylation and degradation by the TRAMP complex and the nuclear exosome, respectively [5, 6]. 

**Figure 1:**
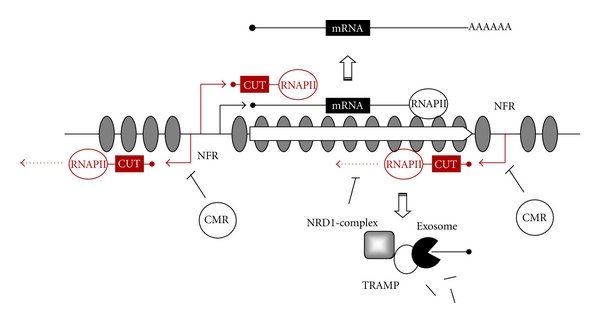
Complexity of the transcriptional landscape in yeast. Transcription of genes encoding stable RNAs by RNAP II is indicated by black lines and cryptic transcription originated from 5′ and 3′ nucleosome free regions (NFRs) is depicted in red. Initiation of transcription is represented by bent arrows and nucleosomes are depicted by grey ovals. Transcription can lead to the production of polyadenylated mRNAs that are competent for export to the cytoplasm and subsequent translation. Initiation of cryptic transcription is minimized by chromatin modifying and remodelling complexes (CMRs) that impose a repressive structure on the chromatin. When those mechanisms are insufficient, the Nrd1 complex terminates transcription and recruits the TRAMP and exosome complexes, which leads to polyadenylation and degradation of the generated CUT.

Work from two independent groups has provided a detailed picture of the genomic distribution of CUTs [7, 8], showing that the vast majority of these transcripts originate from nucleosome-free regions (NFRs) corresponding to promoter regions of *bona fide* genes. Importantly, most of the identified CUTs are transcribed divergently from the promoter region of annotated genes, suggesting that yeast promoters are intrinsically bidirectional. At least two different mechanisms control the intrinsic bidirectionality of promoters, which is a potential source of interference in gene expression. The first acts at the level of chromatin structure and involves different protein complexes implicated in modification of histones or chromatin remodelling that minimize spurious initiation [3, 9]. The second is the Nrd1-dependent termination pathway, mentioned above, which provokes early termination and degradation of the transcripts (Figure 1). In addition to the more frequent antisense CUTs, a non-negligible number of CUTs overlap genes that are transcribed in the same sense. Some of these CUTs have been involved in the regulation of their cognate genes [7, 10–12]. Therefore, CUTs can be by-products of divergent transcription, but also functional units with an important role in the control of gene expression.

Another abundant class of non-coding RNAs has been named SUTs for Stable Unannotated Transcripts [8]. Their origin is the same as for the CUTs (5′ and 3′ end NFRs), but it has been proposed that they differ in their transcription termination mode since they are stable, and thus detectable in wild-type conditions, and often longer than CUTs [1]. However this aspect remains elusive because inactivation of a component of the canonical mRNA termination pathway that depends on the CPF-CF complex (Cleavage and Polyadenylation Factor- Cleavage Factor I) has only a minor effect on the termination of the SUTs tested [13]. A regulatory role for at least a subset of SUTs has also recently been described [14]. Finally, a new category of ncRNAs has been described very recently, which includes mainly antisense transcripts that are stabilized upon mutation of the major cytoplasmic 5′ to 3′ exoribonuclease Xrn1p [15]. These ncRNAs have been designed as XUTs for Xrn1-sensitive Unannotated Transcripts. It has to be noted that often the distinction between XUTs and other ncRNAs is blurry as considerable overlap exists between these three classes of transcripts [7, 8]. A role in repression of gene expression has been proposed for a subset of XUTs and their impact on transcription seems to be more prominent under stress conditions.

Because CUTs are by far the best-characterized class of ncRNAs in terms of origin, metabolism, and implication in regulation of gene expression, in this review we focus on the Nrd1-dependent termination pathway and its key role in limiting pervasive transcription and we describe the mechanisms of regulation that involve expression of a CUT. We also detail other cases of regulation mediated by ncRNAs belonging to other categories as SUTs or XUTs. The impact of cryptic transcription on global gene expression as well as the possible biological significance of this special way of regulation will be discussed.

## 2. Early Termination and Degradation in the Control of Cryptic Transcription

Pervasive transcription constitutes a risk for the cell that is controlled at different levels. Translation of aberrant or defective mRNAs that could result in toxic proteins is prevented by the nonsense-mediated decay (NMD), nonstop decay (NSD), and no-go decay (NGD) pathways in the cytoplasm [16]. However, those pathways act late in the expression process and cannot preclude possible interference of cryptic transcription with normal transcription of genes as, for example, by impeding binding of activator proteins to the promoter region of a downstream gene or by collision with elongating polymerases on a convergent gene [17–19]. Some of these deleterious effects are circumvented by the action of the Nrd1-complex that simultaneously elicits termination early in transcription and recruits the exosome-TRAMP complexes to their target RNAs, facilitating polyadenylation and consequential degradation of the transcripts [20]. The exosome is a conserved multisubunit complex with both endonuclease and 3′–5′ exonuclease activities that functions in degradation of defective transcripts as well as in processing of 3′ ends of stable ncRNAs (snRNAs, snoRNAs, and the 5.8S rRNA). The exosome has a nuclear and a cytoplasmic forms that share a core of ten proteins, being Rrp44p (also named Dis3p) the sole catalytic subunit of the core exosome. The nuclear form of the exosome possesses an additional exonuclease, Rrp6p, that also partakes in the degradation of CUTs (reviewed in [16]). The TRAMP complex has two alternative forms with a common structure, containing a poly-A polymerase (either Trf4p or Trf5p, Trf4p-containing complexes being more abundant), the DexH-box helicase Mtr4p and a zinc-knuckle RNA binding protein (either Air1p or Air2p). In contrast to the protective role of poly-A tails in mRNAs, polyadenylation by the TRAMP complex promotes exosome-mediated degradation, which is thought to be due to the lower processivity of Trf4/5, compared to the mRNA poly-A polymerase [21] and/or the shorter length of the poly-A tails added by the TRAMP complex [22]. The coupled action of the Nrd1-termination complex and the nuclear exosome allows controlling the production of aberrant transcripts at a stage prior to RNA export, possibly avoiding flooding the downstream RNA quality pathways mentioned above (NMD, NGD, *etc.*).

Even though the TRAMP complex and the nuclear exosome are important actors in the control of pervasive transcription, their role has been extensively reviewed elsewhere [23]. We will focus here on the properties of the Nrd1-complex and the data that contribute to elucidate the mechanisms of termination.

## 3. The Nrd1-Nab3-Sen1 Termination Complex 

The Nrd1 complex was first identified for its role in termination and exosome-mediated maturation of sn- and snoRNAs [24]. Subsequently, it was shown to be responsible for termination of the novel class of ncRNAs designed as CUTs [5, 6].

The Nrd1 complex is composed by the RNA binding proteins Nrd1p and Nab3p and the RNA and RNA-DNA helicase Sen1p. Nrd1p is an essential 63 kDa protein that contains an Nab3p interacting region, an RNA recognition motif (RRM) and an N-terminal region that allows interaction with the C-terminal domain (CTD) of the large subunit of RNAP II (CID, CTD Interacting Domain [25, 26]). The CTD of RNAP II consists of tandem repeats of a hepta-peptide (YSPTSPS) that is subjected to different post-translational modifications throughout the transcription cycle and that serves as a landing pad for many proteins involved in key processes such as capping, elongation, termination, and splicing [27]. Phosphorylation at serines 2, 5, and 7 has been shown to be critical for the function and shape of the CTD. Nrd1p interacts preferentially with the Ser5-P form of RNAP II CTD *in vitro* but colocalizes genomewide with the Ser7-P form *in vivo* [26, 28], suggesting an important role of the latter modification. Nab3p is an essential 90 kDa protein that contains an N-terminal domain rich in D and E, a central RRM, and an essential P/Q-rich C-terminal domain of unknown function [29]. It interacts directly with Nrd1p and with Sen1p [29, 30]. Mutational analysis of Nrd1-dependent termination substrates and RNA-binding assays performed with purified RRMs has led to identification of GUAA/G and UCUU as binding sites, respectively, for Nrd1p and Nab3p [31–33]. In addition, *in vivo* RNA-protein crosslinking experiments (CRAC) have recently shown that the preferred binding site for Nab3p *in vivo* is CTTG [22]. Sen1p is an essential 252 kDa protein with a role in termination of ncRNAs as well as several mRNAs [34] that also functions in DNA repair [35, 36]. The first 975 amino acids of Sen1p are dispensable for growth but are involved in the interaction with RNAPII, the RNAse III-like endonuclease Rnt1p, and the nucleotide excision repair endonuclease Rad2p [35], while the C-terminal half contains the essential helicase domain and a motif required for interaction with the Glc7p phosphatase, which dephosphorylates Sen1p *in vitro* [30].

Unlike the canonical mRNA termination pathway that depends on the CPF-CF complex and functions late in transcription, Nrd1-dependent termination is efficient only within a window of less than 1000 bp after transcription initiation, where the RNAP II CTD is phosphorylated mainly at Ser5 and Ser7 [28, 37–40]. 

Although it has been shown that Ser5-P (and possibly Ser7-P) favors Nrd1-dependent termination while Ser2-P antagonizes it [37], the correlation between CTD phosphorylation and termination remains not fully elucidated and the correct levels of each modification appear to be crucial. Consistent with this notion, mutation of proteins involved in the modification of the CTD, such as the Ser2 phosphatase Fcp1p [37], the Ser2 kinase Ctk1p [24], the Ser5 kinase Kin28p, the phosphatase Ssu72p [41, 42], and even the Ser-Pro isomerase Ess1p [43] affects negatively Nrd1-dependent termination. Interestingly, moreover, the role of the CTD and its modifications in termination might not pertain directly to its interaction with Nrd1p because a *nrd1*-ΔCID mutant does not exhibit any detectable termination defect [26].

In contrast to the interaction with the CTD, interaction with the RNA is strictly required for Nrd1-dependent termination [24, 26, 29, 37], although the abundance of the known Nrd1p and Nab3p recognition sites within the different termination substrates is highly variable, ranging from one to more than twelve sites [5, 10]. This variability suggests that additional termination signals yet unidentified might exist. Indeed, recent results obtained in our laboratory have revealed new motifs involved in Nrd1-dependent termination as well as specific arrangement of sites that are required for the termination signals to be functional (Porrua et al., unpublished). The heterogeneity among termination substrates concerns not only the *cis*-acting elements but also the *trans*-acting factors involved in Nrd1-dependent termination. For example, mutations in the catalytic site of the prolyl isomerase Ess1p provoke a defect in termination of a set of snoRNAs but termination of other snoRNAs and a large share of CUTs remain unaffected [43]. In addition, the polyA-binding protein Hrp1p seems to be implicated in termination of some CUTs tested but not others [10]. Furthermore, mutation or deletion of genes encoding proteins involved in histone modification as the histone methyltransferase *SET1* and the histone deacetylase complex Rpd3L exacerbates the termination defects of *NRD1* mutants at most of the CUTs and snoRNAs analyzed but not all [44]. This apparent complexity is maybe the reason why the precise mechanism of Nrd1-dependent termination remains largely not understood. Further work, especially with *in vitro *systems, is needed to fully understand the exact role of each of the proteins involved as well as to dissect the different steps leading to transcription termination.

## 4. Cryptic Transcription in the Control of Gene Expression

Most of the CUTs that function in the regulation of gene expression identified thus far are located upstream or overlap the regulated gene and in the sense orientation. Production of the regulatory CUT normally has a negative impact on transcription of the downstream gene and this effect is exerted in *cis* CUTs can share the TATA box and/or the transcriptional start sites with the regulated gene or can use their owns, implying different mechanisms by which regulation occurs. In this section, we review the best-characterized examples of CUTs implicated in regulation and we briefly comment other types of ncRNAs that also control the expression of genes by different mechanisms.

### 4.1. Regulation by Attenuation

**Figure 2:**
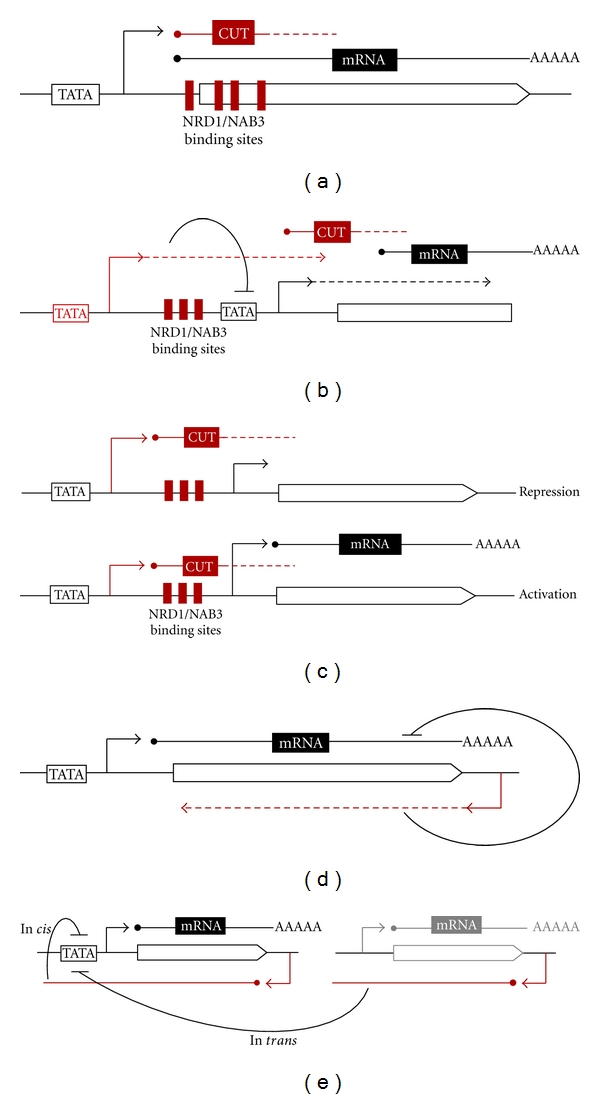
Summary of regulatory mechanisms involving production of ncRNAs Transcription is depicted by dashed arrows, ncRNAs by red lines, and mRNAs by black lines. Transcription start sites (TSSs) are indicated by bent arrows. Red boxes indicate Nrd1-dependent termination signals. (a) *Regulation by attenuation*: a given transcription initiation event can give rise to either an unstable transcript generated by premature termination by the Nrd1 complex or a stable mRNA if transcription is allowed to reach the CPF-dependent terminator. (b) *Regulation by transcriptional interference*: transcription of a CUT (or a stable RNA) occludes the promoter of a downstream mRNA gene thus impairing pre-initiation complex assembly and subsequent expression of the gene. (c) *Regulation by alternative TSS choice*: transcription initiation can occur either at an upstream or a downstream TSS. When the upstream TSS is selected, Nrd1-dependent termination signals are included in the transcript, leading to transcription termination and production of a CUT. When transcription starts at the downstream TSSs, these signals are skipped and a functional mRNA is produced. Regulation occurs at the level of TSS selection. (d) *Regulation by antisense transcription*: antisense transcription impairs the sense of mRNA production without affecting the initiation step. (e) *Regulation by antisense ncRNA*: long ncRNAs are able to act in *cis* and in *trans* to recruit chromatin-modifying enzymes and silence the sense gene. In this case, regulation is mediated by the ncRNA.

The term “attenuation” refers to negative control by a CUT that shares both the TATA box and the transcriptional start sites (TSS) with the regulated gene. After transcription initiation, a fraction of the elongating polymerases is subjected to early termination by the Nrd1-dependent pathway, which generates a non-functional CUT. Only the molecules that escape premature termination can proceed until the CPF-dependent terminator sequences, thus producing a functional mRNA molecule (Figure 2(a)). 

The first and the best-characterized example of attenuation is autoregulation of *NRD1* itself. The *NRD1* transcript behaves both as an mRNA and as a CUT because it contains all the determinants for Nrd1-dependent termination and degradation, as well as the sequences required for normal termination and 3′ end formation by the canonical CPF-dependent pathway. The full-length *NRD1* mRNA is upregulated upon mutation or inactivation of *NRD1*, Nab3p, or *SEN1,* [24, 45]. Nrd1 has a relatively long 5′UTR of about 300 bp that contains Nrd1p and Nab3p binding sites. This sequence is sufficient for triggering Nrd1-dependent termination when inserted into an exogenous gene and provokes a 3- to-4-fold reduction in the levels of *NRD1* mRNA [24]. At least 13 additional Nrd1p- and Nab3p-binding sites are spread within the first 600 bp of *NRD1* coding sequence. Mutation of these motifs individually has a modest, if any, effect on *NRD1* autoregulation, but mutation of all of them in conjunction with modification of the sites in the 5′UTR provokes an additional 2-fold increase of mRNA levels, indicating that sequences within the coding sequence contribute to autorepression [6].

Another gene that shares this mode of regulation is *HRP1*. As in the case of *NRD1*, the *HRP1* 5′UTR is sufficient to trigger early termination when inserted in an exogenous gene. Termination is impaired upon mutation of the components of the Nrd1-complex, but, interestingly, it is also negatively affected by mutation of *HRP1* itself [10, 34]. Hrp1p is an RNA-binding protein that interacts with AU dinucleotide repeats and is implicated in pre-mRNA cleavage and polyadenylation [46] and mRNA export from the nucleus [47]. The work of Kuehner and coauthors suggests that Hrp1p acts in concert with the Nrd1-complex to regulate its own expression and possibly the expression of other genes that are subjected to attenuation.

### 4.2. Regulation by Transcriptional Interference

One of the best examples of transcriptional interference that implicates the production of a CUT relates to the control of the *SER3* gene, whose product catalyses a step in serine byosynthesis, in response to serine availability. The expression of *SER3* is activated in the absence of serine and is repressed under serine-replete conditions by the expression of an upstream CUT designed as *SER3* Regulatory Gene 1 (*SRG1*) [48]. *SRG1* is transcribed from its own promoter and TSS, which are different from those of *SER3, *and it extends up to the first nucleotides of the *SER3* coding region.

In the presence of serine, the sequence-specific activator Cha4p recruits the Swi/Snf chromatin remodeler and the SAGA complex to *SRG1* promoter region. These factors together activate transcription of *SRG1* that subsequently impedes binding of transcription activators and the TATA-binding protein to the *SER3* promoter region [11, 48]. Recent results have shown that the mechanism of transcriptional interference involves the assembly of nucleosomes over the *SER3* promoter region upon transcription of *SRG1*, a process that is mediated by the action of Spt6p and Spt16p, two histone chaperones that facilitate disassembly and reassembly of nucleosomes [49]. In the absence of serine, Cha4p does not interact with Swi/Snf and SAGA complexes, so transcription activation of *SRG1* does not take place and an NFR is formed at the *SER3* promoter region, allowing the binding of sequence-specific activators that are required for full expression of *SER3* (Figure 2(b)).

Termination of *SRG1* transcription occurs at two consecutive sites, being termination at the first site dependent on the Nrd1-complex and termination at the second site presumably dependent on the CPF-pathway. Given that expression of *SRG1* is strong and that each terminator alone might not be sufficiently efficient, the presence of a second terminator could constitute a fail-safe mechanism to prevent the production of a chimeric *SRG1-SER3* transcript. Indeed, depletion of the Nrd1 leads to the accumulation of *SRG1-SER3* RNAs that might eventually give rise to functional Ser3p protein under repressing conditions or to aberrant, potentially toxic translation products [5].

A similar mechanism of transcriptional interference might operate on regulation of *ADH1* and *ADH3* expression in response to zinc-limitation. *ADH1* and *ADH3* encode two different zinc-dependent alcohol dehydrogenases that are repressed during zinc deficiency by the small upstream transcripts *ZRR1* and *ZRR2*, respectively. Expression of the upstream ncRNA is activated by the zinc-responsive regulator Zap1p, and transcription through the *ADH1* and *ADH3* promoter regions prevents binding of the required transcriptional activators [50]. Whether repression involves nucleosome deposition as for *SER3* regulation remains to be assessed. Both transcripts are relatively stable and detectable in a wild-type strain, however, at least part of the termination might be Nrd1-dependent since they are recovered in the CUT fraction in deep sequencing transcriptome analysis [7].

Finally, strong expression of upstream sense CUTs has been detected at some genes involved in glycolysis as *TPI1*, *GPM1,* and *FBA1*. These CUTs are expressed from their own promoter and TSS and are antiregulated relative to their associated mRNA, suggesting that they might be subjected to the same mechanism of transcriptional interference as *SER3* [7].

### 4.3. Alternative Transcriptional Start Site (TSS) Choice

Several genes of the nucleotides biosynthetic pathways *URA2*, *IMD2*, *URA8,* and *ADE12*, involved in synthesis of UTP, GTP, CTP, and ATP, respectively, are regulated by nucleotide availability and are activated when a given nucleotide is missing. All these loci share a similar organization with a 5′ overlapping CUT that is important for regulation (Figure 2(c)). The examples of *IMD2* and *URA2* are the best described [10, 12]. In both cases, expression of the CUT and the mRNA is driven by a common promoter but transcription can start either at an upstream or a downstream site. The region between the two consecutive TSSs contains all the sequence elements required for Nrd1-dependent termination and degradation, so that the use of the upstream TSS leads to the production of a CUT. On the contrary, when transcription starts at the downstream site, the Nrd1 complex termination signals are skipped and the whole coding sequence can be transcribed, leading to the production of a functional mRNA. Selection of the mRNA TSS only occurs under activating conditions (i.e., shortage of the specific nucleotide) while only the CUT TSS is used under nucleotide replete conditions. However, since the common promoter is always active, most of the regulation occurs after preinitiation complex assembly at the step of start site selection.

The presence of the upstream TSS has a clear inhibitory effect on expression of the mRNA under nonactivating conditions, due to the fact that most polymerases never make it to the downstream TSS. Indeed, it has been shown that mutation or deletion of the *IMD2* CUT impairs full repression under guanine replete conditions [51]. Similarly, preventing production of the *URA2* CUT by mutation of its start site leads to expression of *URA2* mRNA even under nonactivating conditions [12]. 

What induces the selection of the downstream TSS upon activation remains, however, not fully clarified. It has been shown that under activating conditions the upstream CUT is still transcribed at the same levels as in repressing conditions in the case of *URA2* and to somewhat lower levels in the case of *IMD2.* Therefore it remains an open question whether the selection of the mRNA TSS occurs because of increased read-through at the upstream CUT TSS or by some independent mechanism. Consistent with the first notion, it has been proposed that under GTP shortage, the upstream TSS is skipped at the *IMD2 *locus because it involves a G as the starting nucleotide thus allowing initiation to the downstream mRNA start site [10]. However, regulation at *URA2* has been shown to be different [12]. In this case, transcription of the CUT always starts at an A (not at U as the previous model would predict) and it is therefore difficult to imagine how UTP shortage could prevent initiation just based on nucleotide availability. Rather, it has been shown that activation of the mRNA sites requires a T-rich sequence comprised in the region between the CUT and the mRNA start sites and thus transcribed only at the CUT level [12]. This regulatory mechanism has revealed to be extremely complex and might be considerably different from one system to another. Additional work needs to be done on the different models to clarify the aspects that remain elusive. For example, it is unclear thus far whether, in addition to its repressive role under nonactivating conditions, expression of the upstream CUT plays any active role in selection of the downstream TSS upon nucleotide shortage [12]. However, it is clear that the Nrd1-dependent termination-degradation pathway plays an essential role in diverting the constitutive expression of a gene to a nonfunctional pathway, terminating and degrading transcripts initiated under conditions that do not require expression of the gene. 

### 4.4. Other Ways of Regulation Involving an ncRNA

In this section, we include the most relevant cases of gene regulation that are mediated by other types of ncRNAs whose production is in principle independent of the Nrd1p pathway.

As we mentioned before, a second abundant class of ncRNAs includes stable transcripts that are originated by 5′ and 3′ NFRs as the CUTs and receive the name of SUTs. A recent genomewide study has shown that around 5% of genes overlap an SUT that is transcribed in the opposite strand and extends beyond the TSS of the gene. This set of ORFs is enriched in stress response and environment-specific genes and exhibits a larger expression dynamic range upon environmental changes, although identical maximal levels of expression, than genes with other configurations. The authors propose that in most cases, transcription of the antisense SUT represses expression of the sense gene under nonactivating conditions, which more efficiently locks the gene in an off-state under nonactivating conditions [14]. In addition to this possible general role of antisense transcription, condition-specific repression of a gene by an antisense ncRNA has been described in a number of cases. These sense-antisense modules can act through different mechanisms. They can act either in *cis* or in *trans* and several of them have been shown to be physiologically regulated as it is the case for the *PHO84*, *IME4*, *ZIP2,* or the *GAL1-GAL10* loci.

*IME4* and *ZIP2* are two genes specifically expressed during meiosis and repressed in haploids by the cognate antisense ncRNAs *RME2* and *RME3,* respectively [18, 52]. These antisense transcripts provide a sophisticated way to activate genes as they are transcriptionally repressed in diploids by the a1p/*α*2p heterodimer, allowing expression of the meiotic genes. Control by *RME2* and *RME3* only works in *cis *and *via *a transcriptional interference-like mechanism. However, *RME2* and *RME3* ncRNAs do not need to extend until the promoter region of *IME4* and *ZIP2* to exert their inhibitory function and TBP is always present in the 5′ of the sense genes *IME4* and *ZIP2*, even under repressive conditions. This suggests that the antisense RNA does not prevent formation of a preinitiation complex upstream of the sense gene but acts later, possibly by preventing elongation of the sense RNA (Figure 2(d)).

The *GAL1*-*GAL10* locus provides another example of *cis*-regulation by an ncRNA. At this locus, a transcript antisense to *GAL10* is generated only in glucose while *GAL1* and *GAL10* are silent whereas it becomes undetectable in galactose media when *GAL1* and *GAL10* are active. This antisense RNA allows full repression of the *GAL* locus in low glucose conditions presumably by controlling the recruitment of chromatin remodelling enzymes such as the histone methyltransferase Set1p and the histone deacetylase complex Rpd3S [53, 54].

The *PHO84* gene is also controlled by an antisense RNA. The ncRNA represses *PHO84* transcription through different mechanisms as it acts not only in *cis* but also in *trans* and in both cases, production of a long antisense RNA spanning the *PHO84* gene until its UAS sequence is necessary (Figure 2(e)). *PHO84* is regulated in ageing cells, when the antisense RNA is stabilized thus turning off *PHO84* expression. *cis-* antisense-mediated silencing requires recruitment of the histone deacetylase Hda1/2/3 which de-acetylates histones at the *PHO84* promoter locus [55]. The endogenous copy of *PHO84* can also be silenced *in trans *by production of the antisense RNA from a plasmid, but in this case the silencing does not involve Hda1/2/3 [56]. 

The Ty1 transposon is also partially regulated by an antisense unstable transcript. This antisense RNA acts in *trans* to repress both mobility and expression of Ty1 [54]. The Ty1 antisense ncRNA is an XUT, and in a very recent work from van Dijk et al. [15], the authors propose that, as in the case of Ty1, many other genes are transcriptionally repressed by their antisense XUTs by a mechanism of silencing dependent on the action of the methyltransferase Set1p. Since repression is observed upon inactivation of the cytoplasmic RNAse Xrn1p, and subsequent stabilization of XUTs, an important mechanistic aspect that remains to be elucidated is how these cytoplasmic RNAs manage to exert their repressive role in the nucleus. 

Additional data support a link between noncoding transcription and silencing [57, 58]. However, in contrast to the regulatory systems mentioned above where an ncRNA promotes silencing, here stabilization of an ncRNA derived from heterochromatic rDNA spacer region counteracts silencing through modifications in the structure of chromatin. Nonetheless, whether this effect on silencing is mediated by transcription itself or by the ncRNA is still unclear.

Finally, a different example of regulation is that of the *PHO5* locus, where transcription of an ncRNA antisense to *PHO5* is necessary for nucleosome eviction at the promoter, which is required for transcriptional activation of the gene [59]. Thus, in this case the ncRNA plays a positive role in transcription, in contrast to the previously mentioned ncRNAs, which impact negatively the expression of the cognate genes.

## 5. Concluding Remarks

Cryptic transcription is widespread in yeast. Recent genomewide analyses of *Saccharomyces cerevisiae* transcriptome have revealed more than 1400 CUTs/SUTs generated by NFRs, mainly at promoter regions of *bona fide* genes, either sense or antisense to the associated gene [7, 8]. These studies have, however, been performed only in a few physiological conditions (exponential growth supported by a limited number of carbon sources) and upon mutation of only some of the components of the exosome and TRAMP complexes. Therefore, it is possible that many additional ncRNAs are produced under different conditions or in different growth phases. Consistently, a number of new unannotated transcripts have been detected upon deletion of the RNA exonuclease Xrn1p [15]. In addition, a recent analysis of the yeast transcriptome during sporulation has revealed the presence of new ncRNAs specific of the meiotic phase designed as MUTs for Meiotic Unannotated Transcripts [60]. Furthermore, several additional CUTs have been identified upon mutation of the catalytic subunit of the core exosome Dis3p (Gudipati et al., unpublished results). In addition to ncRNAs originated at 5′ and 3′ NFRs, cryptic transcription can occur at regions internal to genes upon mutation of the histone chaperones Spt6p and Spt16p [61], as well as the histone deacetylase complex Rpd3S and the histone methyltransferase Set2p [62], presumably because of the formation of transitory NFR in coding regions that can unveil cryptic promoters. This is in agreement with the notion that the chromatin structure plays an important role in the control of cryptic transcription. 

The occurrence of such a high level of cryptic transcription might imply that transcription initiation by eukaryotic RNAPII can occur with relatively low specificity in the absence of active mechanisms to prevent it. This behavior is rather different from that of the prokaryotic RNA polymerase, which always binds motifs that are quite conserved and initiates transcription at a defined distance downstream from the promoter sequences [63]. In many ways, this intrinsic “promiscuity” of the eukaryotic RNAPII constitutes a disadvantage as it requires efficient pathways to (i) counteract the potentially deleterious effects of pervasive transcription on the expression of functional transcripts (e.g., the Nrd1-dependent termination pathway) and (ii) degrade a large amount of nonfunctional or aberrant RNAs that could impact negatively the physiology of the cell, for example, by sequestering the export and translation machineries or by producing toxic protein products upon translation (e.g., nuclear and cytoplasmic RNA quality control pathways). In spite of these drawbacks, the maintenance of such flexibility might imply some evolutionary and/or functional advantages. For instance, it is possible that the production of a plethora of different ncRNAs allows some of them to evolve towards functional molecules, which would then be stabilized and confer higher fitness to the cell. In addition, a high flexibility in transcription initiation provides multiple opportunities for regulation, enabling the development of sophisticated regulatory mechanisms as those reviewed here. 

The expression of the ncRNA normally plays a repressive role on transcription of the cognate gene. In general, genes that are subjected to both positive and negative control have a higher dynamic range of expression, which allows a fine-tuned response to environmental conditions [14]. In addition, active repression under nonactivating conditions minimizes “leakyness” in gene expression, avoiding production of proteins when they are not required by the cell and/or waste of energy. A remaining question is why the relatively complex negative control involving production of an ncRNA should be more advantageous than a classical regulation system based on protein repressors. The answer is not always obvious. In the case of regulation by attenuation, where the Nrd1-complex elicits partial premature termination, this mechanism provides an efficient way for the proteins involved in termination to control their own production by a feed-back loop. The mechanism of transcriptional interference might allow transcriptional activators to be turned into repressors using the same molecular mechanism by which they both promote expression of the target genes and transcription of an ncRNA that prevents expression of the repressed genes. Finally, the regulation by alternative TSS selection seems to be associated to particular metabolic pathways (i.e., nucleotides biosynthesis) that require a fast response to environmental changes because they lead to the production of key molecules whose shortage would impact many cellular processes. A promoter that is already activated but not physiologically functional because it leads to the production of a CUT is more susceptible to be diverted to a functional state in the presence of the appropriate signal because it is endowed with the proper chromatin structure and a repertory of transcription factors and RNAPIIs. In that sense, production of the CUT would enable a “preactivated” state of the promoter, so that it would be ready for a fast response to changes in the intracellular environment.

Among the hundreds of ncRNAs that are associated to *bona fide *genes, only a few have been studied thus far and it is likely that expression of many more is modulated by the presence of an ncRNA and that additional regulatory mechanisms exist. Much more experimental work is needed to evaluate the global impact of cryptic transcription on gene expression and to unveil the multiple associated mechanisms of regulation.

In conclusion, this paper is focused on the main progresses in the study of ncRNAs done in the model organism *Saccharomyces cerevisiae* over the last years. However, the phenomenon of cryptic transcription and the regulation of gene expression by ncRNAs are conserved from yeast to animals. The most relevant works concerning the characterization of the noncoding transcriptome and the multiple categories of ncRNAs, as well as their associated regulatory functions, present in higher eukaryotes are nicely detailed in other recent reviews [1, 2, 64].
